# Non-target Site Tolerance Mechanisms Describe Tolerance to Glyphosate in *Avena sterilis*

**DOI:** 10.3389/fpls.2016.01220

**Published:** 2016-08-12

**Authors:** Pablo T. Fernández-Moreno, Ricardo Alcantara-de la Cruz, Hugo E. Cruz-Hipólito, Antonia M. Rojano-Delgado, Ilias Travlos, Rafael De Prado

**Affiliations:** ^1^Agricultural Chemistry and Soil Sciences, University of CórdobaCordoba, Spain; ^2^Bayer CropScienceMexico, Mexico; ^3^Faculty of Crop Science, Agricultural University of AthensAthens, Greece

**Keywords:** *Avena sterilis*, glyphosate-tolerant, NTSR/NTST, TSR

## Abstract

Sterile wild oat (*Avena sterilis* L.) is an autogamous grass established in warm climate regions. This species has been used as a cover crop in Mediterranean perennial crops during the spring period prior to initiating competition with the main crop for water and nutrients. However, such cover crops need to be controlled (by glyphosate or tillage) before the beginning of summer period (due to the possibility of intense drought stress). In 2011, the olive grove farmers of southern Spain expressed dissatisfaction because of the ineffective control with glyphosate on *A. sterilis*. Experiments were conducted to determine whether the continued use of glyphosate over a 5 year period had selected a new resistant or tolerant species. The GR_50_ values obtained for *A. sterilis* were 297.12 and 245.23 g ae ha^−1^ for exposed (E) and un-exposed (UE) glyphosate accessions, respectively. The spray retention and shikimic acid accumulation exhibited a non-significant difference between the two accessions. The results of ^14^C- glyphosate absorption was the same in the two accessions (E and UE), while the translocation from the treated leaf to the rest of the shoots and roots was similar in *A. sterilis* accessions. Glyphosate metabolism to aminomethylphosphonic acid (AMPA) and glyoxylate was similar in both accessions, but increased after treatment with glyphosate, indicating that metabolism plays an important role in tolerance. Both *A. sterilis* accessions, present similarity in the 5-enolpyruvylshikimate-3-phosphate synthase (EPSPS) activity enzyme with different glyphosate concentrations and without glyphosate, confirming that both accessions present the same genomic characteristics. The above-mentioned results indicate that innate tolerance to glyphosate in *A. sterilis* is probably and partly due to reduced herbicide absorption and translocation and metabolism compared to the susceptibility of other grasses weeds like *Chloris inflata, Eleusine indica*, and *Lolium rigidum*.

## Introduction

Wild oats are known as *Avena fatua* L. or various subspecies of *A. sterilis* L. The best known subspecies are *A. ludoviciana* Dur. (*A. sterilis* ssp. *ludoviciana*) and *A. sterilis* (*A. sterilis* ssp. *sterilis*, also known as *A. macrocarpa* Moench) (Chancellor, 1976). *A. sterilis* is an autogamous grass species, and therefore its isolated populations can produce seeds. In the absence of competition, this weed is characterized by enormous seed production of more than 400 seeds per plant. *A. fatua* is typical of temperate regions of northwestern Europe, North America, South America (Argentina and Uruguay), Australia, and South Africa. The subspecies of *A. sterilis* have been mostly established in Mediterranean climate regions, such as southern Europe, and North Africa (Travlos and Giannopolitis, 2010).

Over the last 15 years, these grasses have been used as cover crops in Mediterranean perennial crops, such as olive groves (*Olea europaea* L.), citrus groves (*Citrus* spp.), and vineyards (*Vitis vinifera* L.). These cover crops have helped to reduce the soil erosion in these areas. Currently, seed companies have selected species that show an early maturing, a low or no regrowth capacity and a high persistence of stubble, and therefore, these species protect the soil during unfavorable periods. Such species are very competitive and can be maintained for several years without the need of sowing every year. However, during the spring period, previous to starting their competition with the main crop for water and nutrients, the weeds have to be controlled by chemical or mechanical control. Since the adoption of cover crops by farmers, glyphosate has been the most widely used herbicide due to its high efficacy on weeds, both monocotyledonous and dicotyledonous ones (Franz et al., 1997). Glyphosate is the only herbicide that acts by inhibiting the enzyme 5-enolpyruvylshikimate-3 phosphate synthase (EPSPS, EC 2.5.1.19), and prevents the biosynthesis of three aromatic amino acids: phenylalanine, tyrosine and tryptophan (Geiger and Fuchs, 2002). However, the extended use of glyphosate over the last 20 years without performing an integrated weed management with rotation of herbicides and adoption of other cultural practices, has led to the selection of resistant plants (Sammons and Gaines, 2014). Moreover, such practices have progressively changed the composition of the weed flora in olive groves, orchards and vineyards (Puricelli and Tuesca, 2005).

It has to be noted that in many cases there is still misunderstanding and confusion between the terms “resistance” and “tolerance.” Tolerance is “the inherent capacity of a species to survive and reproduce after herbicide treatment at field doses” [Weed Science Society of America (WSSA), 1998]. This implies that there was no genetic manipulation or selection to achieve herbicide tolerance, it is naturally an innate tolerant plant.” Resistance is “the ability of a plant to survive and reproduce following exposure to herbicide doses normally lethal to the wild type. In a plant, resistance may be natural or induced by techniques such as genetic engineering or selection of variants produced by tissue culture or mutagenesis” (Beckie, 2006). Although well-defined in this way, the difference between evolved resistance and natural tolerance is not always well perceived by growers (Owen, 2008; Dubois and Plancke, 2010).

The selection of resistance/tolerance to glyphosate is due to two mechanism groups known as non-target site resistance/tolerance (NTSR/NTST) and target site resistance (TSR). The NTSR/NTST involves a reduced rate of herbicide in the meristem tissues due to limited absorption/translocation and/or sequestration of the herbicide into compartments as vacuoles. Metabolic pathways capable of degrading the herbicide to non-toxic compounds in plants also belong to this group. Innate tolerance acquired has been attributed to NTSR/NTST mechanisms (De Prado and Franco, 2004; Cruz-Hipólito et al., 2009, 2011; Sammons and Gaines, 2014). However, resistance has been reported for both group TSR and NTSR mechanisms, with TSR being more common than NTSR. TSR has been produced by one or more mutations in the DNA sequence (Sammons and Gaines, 2014; Yu et al., 2015), or by the overexpression of the EPSPS protein by gene amplification (Gaines et al., 2010; Salas et al., 2012, 2015).

In 2011, the olive grove farmers of southern Spain expressed complains on the inadequate efficacy of glyphosate on *A. sterilis*. The common practice by farmers was the application of glyphosate 2–3 times per year for over 5 years, without any application of herbicides with different mode of action.

The main objective of the present study was to determine whether the continued use of glyphosate over a 5 year period had selected a new resistant or a tolerant accession. This was achieved by means of surveys and collection of *A. sterilis* by different provinces in southern Spain and after that by conducting physiological and molecular studies on the mechanism(s) that could probably endow innate tolerance to glyphosate.

## Materials and methods

### Plant material and growing conditions

In the summer of 2012, sterile oat (*A. sterilis*) seeds were harvested from an olive grove in the province of Antequera (Malaga, southern Spain). Over the last 5 years, this specific population had been exposed to glyphosate applications of 900 g ae ha^−1^ (Roundup Energy® 45% w/v, SL, Monsanto Spain). Seeds from this field were called E (seeds exposed to glyphosate applications). Furthermore, seeds of *A. sterilis* which had never been exposed to glyphosate treatments, were collected from different areas of southern Spain and recorded as UE (never exposed to glyphosate). In particular, samplings were conducted in the provinces of Jaén (UE01-UE06 from olive groves), Córdoba (UE07-UE10 from vineyards; UE11-UE17 from citrus orchards), Huelva (UE18-UE20 from citrus orchards), and Sevilla (UE21-UE22 from olive groves).

In addition, three grass weed populations commonly distributed in the perennial crops (*Chloris inflata, Eleusine indica*, and *L. rigidum*) which had never been exposed to glyphosate (UE) were also used. All seeds were germinated in Petri dishes on filter paper moistened with distilled water and placed in a growth chamber at 28/18°C (day/night) with a photoperiod of 16 h, 850 μmol m^−2^ s^−1^ photosynthetic photon flux and 80% relative humidity.

Seedlings of the plants in the stage of two leaves (BBCH 12) were transplanted into pots containing sand/peat in a 1:1 (v/v) ratio, placed in the growth chamber under the same conditions as previously described.

### Dose-response assay

Glyphosate applications were made at the BBCH 13-14 stage (Hess et al., 1997), with a laboratory spray chamber (SBS-060 DeVries Manufacturing, Hollandale, MN) equipped with TeeJet 8002 flat fan nozzles (Spraying Systems Co., Wheaton, IL) delivering 200 L ha^−1^ at 200 kPa at a height of 50 cm. The following glyphosate (Roundup Energy SL, 450 g ae L^−1^ as isopropylamine salt, Monsanto) rates were used: 0, 31.25, 62.5, 125, 250, 500, 1000, and 2000 g ae ha^−1^. The experiment was arranged in a completely randomized design with six replicates of each rate. The experiment was repeated three times. Plants were harvested 30 days after treatment (DAT) and immediately weighed to determine fresh weight.

### Spray retention assays

At stage BBCH 13-14, E, and UE accessions of *A. sterilis* were sprayed with 300 g ae ha^−1^ of glyphosate and 100 mg L^−1^ Na-fluorescein (Fluoroscein-sodium indicator, CI 45350 K37052987 724, Merck, Darmstadt, Germany) under the conditions described above. Once the foliage had dried (20 to 25 min), shoot tissues were cut at ground level. Each tissue was immersed in 50 mL of 5 mM NaOH for 30 s to remove spray solution. Fluorescein absorbance was determined using a spectrofluorometer (Hitachi F-2500, Tokyo, Japan) with an excitation wavelength of 490 nm and absorbance at 510 nm. Dry biomass of plant tissue was recorded following exposure to 60°C for 48 h. The experimental design was completely randomized with four replicates, where each replicate included three plants of each accession. Spray retention was expressed as mL of spraying solution per g of dry matter.

### Whole plant shikimate accumulation assay

Plants from both E and UE accessions of *A. sterilis* and two populations of *L. rigidum*, one glyphosate resistant (R) and one susceptible (S) as previously confirmed by Fernández et al. (2015) were treated with glyphosate at 300 g ae ha^−1^ with the laboratory spray chamber under conditions described above at the BBCH 13-14 stage. At 12, 24, 48, 72, and 96 h after treatment (HAT), 50 mg of treated and non-treated plant tissue was harvested, homogenized and placed in a vial containing 1 mL of 1 M HCl and then immediately frozen in liquid nitrogen. The shikimic acid accumulation was determined according to Singh and Shaner (1998). Absorbance was measured with a spectrophotometer (Beckman DU-640, Fullerton, CA) at 380 nm. The net shikimic acid accumulation was deduced from the difference between treated and non-treated plants. The experiment was performed in triplicate on five treated and five non-treated plants of *A. sterilis* and *L. rigidum*, and the results were expressed as μg per g of fresh weight. The rate of shikimic acid accumulation (μg g^−1^ fresh weight h^−1^) was measured between 12 and 96 HAT.

### Leaf segment shikimate accumulation assay

Leaf segments (50 mm diameter) were harvested from the youngest fully expanded leaf from a pull of 15 plants per E and UE accessions of *A. sterilis* and R and S glyphosate population of *L. rigidum* at the 3–4 tiller stage (Hanson et al., 2009). Approximately 50 mg of fresh tissue was transferred to 2 mL eppendorfs containing 1 mL of 1 mM NH_4_H_2_PO_4_ (pH 4.4). Glyphosate was added to eppendorfs at following concentrations: 0, 0.1, 0.5, 1, 5, 10, 50, 100, 200, 400, 500, 600, and 1000 μM. The eppendorfs were incubated in a growth chamber during 24 h under the same conditions as during plant growth. After 24 h, the eppendorfs stored at −20°C until analysis. Eppendorfs were removed from the freezer and thawed at 60°C for 30 min. 250 μL of 1.25 N HCL were added to each eppendorf, and placed at 60°C for 15 min. A 125 μL aliquot from each eppendorf was pipetted into a new 2 mL eppendorf, and 500 μL of periodic acid and sodium metaperiodate (0.25% [w/v] each) was added. They were incubated at room temperature for 90 min, after which 500 μL of 0.6 N sodium hydroxide and 0.22 M sodium sulfite was added. All eppendorfs were transferred to glass vials. Samples were measured in a spectrophotometer at 380 nm within 30 min. Each glyphosate concentration contained three replicates of each accession. The assay was repeated twice.

### ^14^C glyphosate absorption and translocation

Absorption and translocation study was carried out following the methodology proposed by Michitte et al. (2007). The ^14^C glyphosate was mixed with commercially formulated glyphosate to prepare a solution with a specific activity of 0.834 KBq μL^−1^ and a glyphosate concentration of 1 g ae L^−1^ (300 g ae ha^−1^ in 300 L). Plants of both accessions in BBCH 14-15 stage were treated with the radiolabeled herbicide by applying one droplet of 1 μL of glyphosate solution (0.834 KBq μL^−1^) on the adaxial surface of the second leaf in each plant using a micropipette (LabMate). The ^14^C glyphosate unabsorbed in the treated leaf was removed with 3 mL of water: acetone solution (1:1, v/v) after 12, 24, 48, 72, 96, and 144 h after droplet application. Preliminary assays with both accessions studied had revealed that the glyphosate absorption leveled-off at 96 h after the droplet applications. The rinsate was mixed with 2 mL of scintillation spectrometry (LSS) (Scintillation Counter, Beckman LS 6500, Fullerton CA). The plants were separated into the treated leaf, rest of the shoot and root after being placed in cellulose cones. The plant tissue was dried at 60°C for 96 h and combusted in a biological sample oxidizer (Packard Tri Carb 307, Perkin-Elmer, Waltham, MA). The ^14^CO_2_ evolved was trapped and counted in 18 mL of a mixture of Carbo-Sarb E and Permafluor (9:9, v/v) (Perkin-Elmer). Thus, over 95% of the total radioactivity applied was recovered. There were five replicates (each one with three plants) and the experiment was arranged in a completely randomized design. The proportion of absorbed herbicide was expressed as [% absorbed = (KBq in combusted tissue/KBq in combusted tissue + KBq in leaf washes) × 100].

### ^14^C glyphosate visualization

^14^C glyphosate translocation was visualized in *A. sterilis* E and UE accessions, following the method proposed by Fernández et al. (2015). Each accession was treated and collected in the same way as described in the absorption and translocation assays. The whole plants were rinsed, pressed and then left to dry at room temperature for four days. Then, the dried plants were placed on a 25 × 12.5 cm phosphor storage film for 14 h and scanned for radiolabel dispersion on a phosphor imager (Cyclone, Perkin-Elmer). The experiment was carried out with three plants per accession (E and UE).

### Metabolism study

Plants of E and UE accessions were treated with a glyphosate rate of 300 g ae ha^−1^ at BBCH 13-14 stage. At 12, 24, 48, 96, and 144 HAT, glyphosate and its metabolites, i.e., AMPA (aminomethylphosphonic acid), glyoxylate, and sarcosine were determined by reversed-polarity capillary electrophoresis following the methodology described by Rojano-Delgado et al. (2010). The calibration equations were established using non-treated plants and known concentrations of glyphosate and its metabolites, which were determined from their enclosed areas under the peaks in the electropherogram. The average value for the amount of glyoxylate naturally produced by the plant was subtracted from the average of the produced or reduced amount after treatment of each accession (De Carvalho et al., 2012). The experiment was arranged in a completely randomized design with four plants per accession and repeated three times.

### EPSPS enzyme activity assays

The enzyme extraction was conducted according to the protocol described by Sammons et al. (2007). Five gram of the leaf tissue of E and UE accessions of *A. sterilis*, and R and S populations of *L. rigidum* plants were ground to fine powder in a chilled mortar. Immediately after that, the powdered tissues were transferred to tubes containing 100 mL of cold extraction buffer (100 mM MOPS, 5 mM EDTA, 10% glycerol, 50 mM KCl, and 0.5 mM benzamidine) containing 70 μL of β-mercaptoethanol and 1% in polyvinylpolypyrrolidone (PVPP). Samples were previously stirred and subsequently centrifuged for 40 min (18,000 g) at 4°C. The supernatant was decanted though into a beaker using a cheesecloth. (NH_4_)_2_SO_4_ was added to the solution to obtain 45% (w/v) concentration, with stirring during 30 min. After that, the mix was centrifuged at 20,000 g for 30 min at 4°C. The previous step was repeated to precipitate the protein in the extracts but in that case with a (NH_4_)_2_SO_4_ concentration of 80% (w/v) stirring for 30 min. Finally they were centrifuged at 20000 × g for 30 min at 4°C.

All the pellets were dissolved in 3 mL of extraction buffer and dialyzed in 2 L of dialysis buffer (30 mm, 1000-MWC dialysis tubing at 4°C on a stir plate) over 12 h. The protein concentrations were determined by Bradford assay.

The assay for the determination of EPSPS activity followed the methodology described by Dayan et al. (2015) using the EnzCheck® phosphate assay Kit (Invitrogen, Carlsbad, CA) for determinate the inorganic phosphate release. The EPSPS activity from accessions and populations was determined in the presence and absence of glyphosate. The glyphosate concentrations used were: 0, 0.1, 1, 10, 100, and 1000 μM to determine the enzyme activity inhibition. The used assays buffer was composed of 1 mM MgCl_2_, 10% glycerol, and 100 mM MOPS, 2 mM sodium molybdate, and 200 mM NaF. The experiment was repeated three times for all samples. EPSPS enzyme activity was expressed as percentage of enzyme activity in presence of glyphosate respect to the control (without glyphosate).

### Statistical analyses

Dose-response data were subjected to non-linear regression analysis using a four-parameter log-logistic equation (Equation 1) to determine the glyphosate dose causing 50% reduction in fresh weight (GR_50_), the dose causing 50% mortality (LD_50_), the herbicide rate 50% inhibition of EPSPS activity (I_50_) compared with the untreated control (Alarcón-Reverte et al., 2015; Fernández et al., 2015). Regression analysis was conducted using the statistical freeware program R 3.2.4 with the *drc* package (Ritz et al., 2015).

(1) $$\begin{array}{l} \text{Y}=\left(\left[\left(\text{d}-\text{c}\right)/1+{\left(\text{x}/\text{g}\right)}^{\wedge }\text{b}\right]\right)+\text{c} \end{array}$$

where Y is the above ground fresh weight or the survival expressed as the percentage of the non-treated control, c and d are the coefficients corresponding to the lower and upper asymptotes, b is the slope of the line, g the herbicide rate at the inflection point halfway between the upper and lower asymptotes (GR_50_, LD_50_, I_50_), and x (independent variable) is the herbicide rate.

Resistance Indices (R.I.) was calculated as E-to-UE GR_50_ or LD_50_ ratios. Other indices called Tolerance Indices (T.I.) was calculated between UE accession of *A. sterilis* and UE grass weed populations. Values of GR_50_, LD_50_, and I_50_ were considered to be statistical different when their respective R.I. or T.I. ratios differed from 1 at α = 0.05. A non-significant lack-of-fit test (*P* = 0.9568) indicated that the dose-response data were well described by the selected model. The rate of shikimic acid accumulation was obtained by linear regression.

Treatment means, where appropriate, were separated using Tukey's honestly significant difference (HSD) at α = 0.05. Inspection of error distributions and scatter plots among variables suggested that assumptions of linearity and normality held reasonably well for all analyses. ANOVA was conducted using Statistix (version 9.0; Analytical Software, USA).

## Results

### Dose-response assays

**Figure 1:**
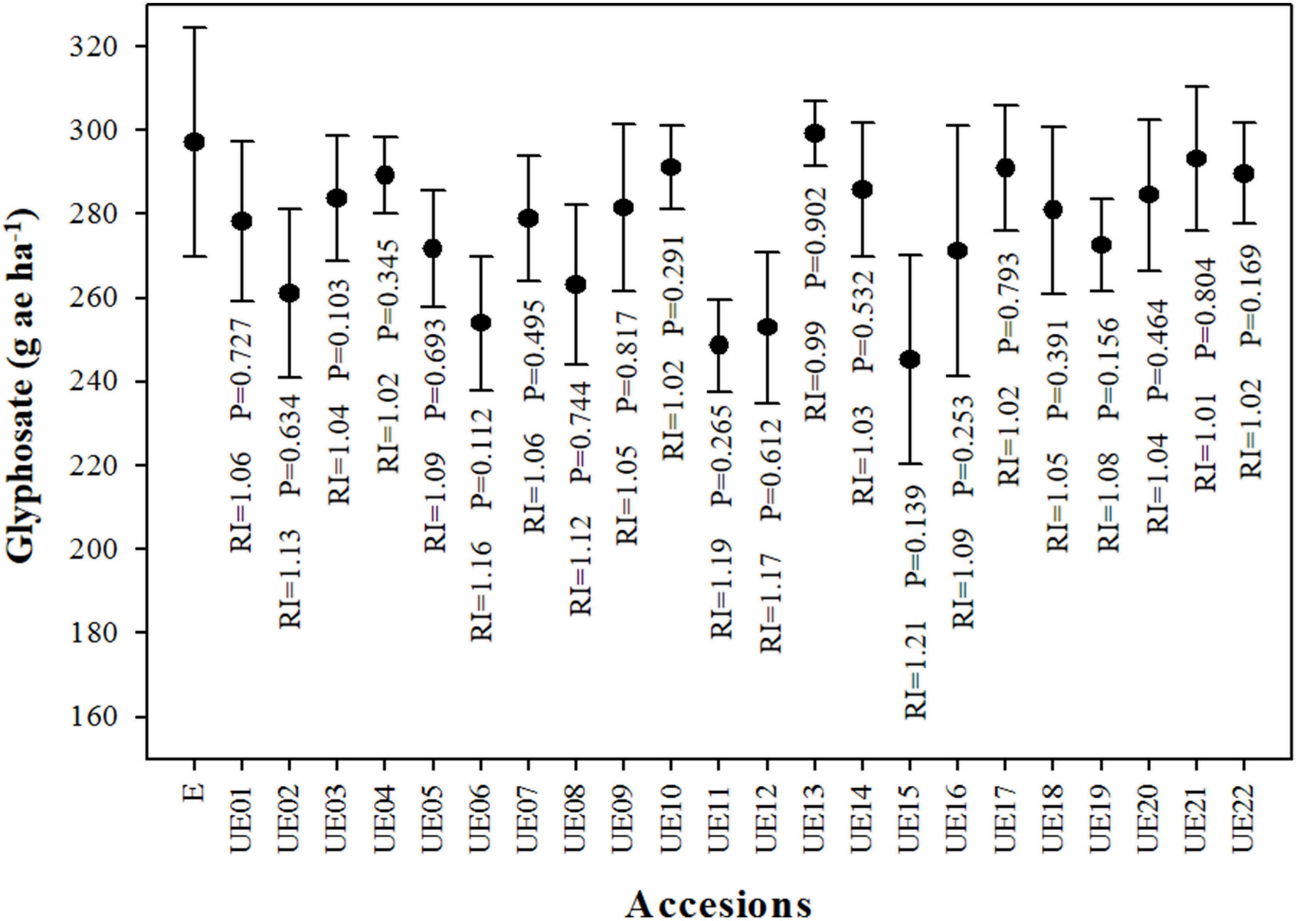
**Fresh weight reduction 50% (GR_50_) in 23 accessions of *A. sterilis* (1 E accession, and 22 UE one) from different provinces of southern Spain**. Vertical bars correspond to standard errors. P = *p*-value. RI (Resistance Index) = GR_50_ (E)/GR_50_ (UE).

Dose-response assays of 23 accessions (E, and UE01 to UE22) of *A. sterilis* resulted to similar GR_50_ values for all accessions. It has to be noted that there was only a slight difference of 51.89 g ae ha^−1^ between the E accession and the lowest GR_50_ value, but it showed no significant differences (*P* = 0.139) (Figure 1, Table 1). Due to these GR_50_ values, it was only chosen one accession with the lower value among all obtained. In particular, the UE15 accession was chosen and showed a GR_50_ value of 245.23 g ae ha^−1^. For all the following assays, this accession was used and hereafter referred to as UE.

**Table 1:** **Estimated parameters for the response curves of accessions of *A. sterilis* and three susceptible grass weed populations treated with glyphosate**.

**Species**		**GR_50_ (g ae ha^−1^) (95% CI)^a^**	**R. I**.	**T. I**.	***P***
*A. sterilis*	E	297.12 (324.40–269.84)	1.21		0.139
	UE	245.23 (270.12–220.34)			
*C. inflata*	UE	57.23 (64.65–49.81)		4.28	<0.0001
*E. indica*	UE	82.05 (90.51–73.59)		2.98	<0.0001
*L. rigidum*	UE	98.12 (105.39–90.85)		2.49	<0.0001
**Species**		**LD_50_ (g ae ha^−1^)(95% CI)**	**R.I.**	**T.I.**	***P***
*A. sterilis*	E	527.64 (565.61–489.67)	1.06		0.384
	UE	494.39 (526.16–462.62)			
*C. inflata*	UE	86.49 (91.36–81.62)		5.71	<0.0001
*E. indica*	UE	109.46 (116.08–102.84)		4.51	<0.0001
*L. rigidum*	UE	137.21 (142.94–131.48)		3.60	<0.0001

E, exposed to glyphosate applications; UE, never exposed to glyphosate applications.

R. I. (Resistance Indices), GR_50_ or LD_50_ (E)/GR_50_ or LD_50_ (UE). T. I. (Tolerance Indices), GR_50_ or LD_50_ (UE A. sterilis)/GR_50_ or LD_50_ (UE).

a CI values are the 95% confidence intervals (n = 6).

Table 1 shows the GR_50_ and LD_50_ values obtained for both accessions of *A. sterilis*. The GR_50_ value for E accession was 297.12 g ae ha^−1^. R.I. value obtained was 1.21. Assays performed on three susceptible glyphosate grass weed populations (*C. inflata, E. indica*, and *L. rigidum*) showed GR_50_ values between 57.27 (*C. inflata*) and 98.12 g ae ha^−1^ (*L. rigidum*). The results regarding Tolerance Indices indicated that UE accession of *A. sterilis* was 2.49 to 4.28 times more tolerant to glyphosate than the other populations (Table 1). The LD_50_ values were also similar to those observed for GR_50_ values (Table 1).

### Spray retention

Our results were 0.71 ± 0.13 and 0.72 ± 0.25 mL of sprayed solution retained per g dry weight for E and UE accessions, respectively. These results indicated no significant differences between accessions (*P* = 0.898).

### Shikimic acid accumulation assays

**Figure 2:**
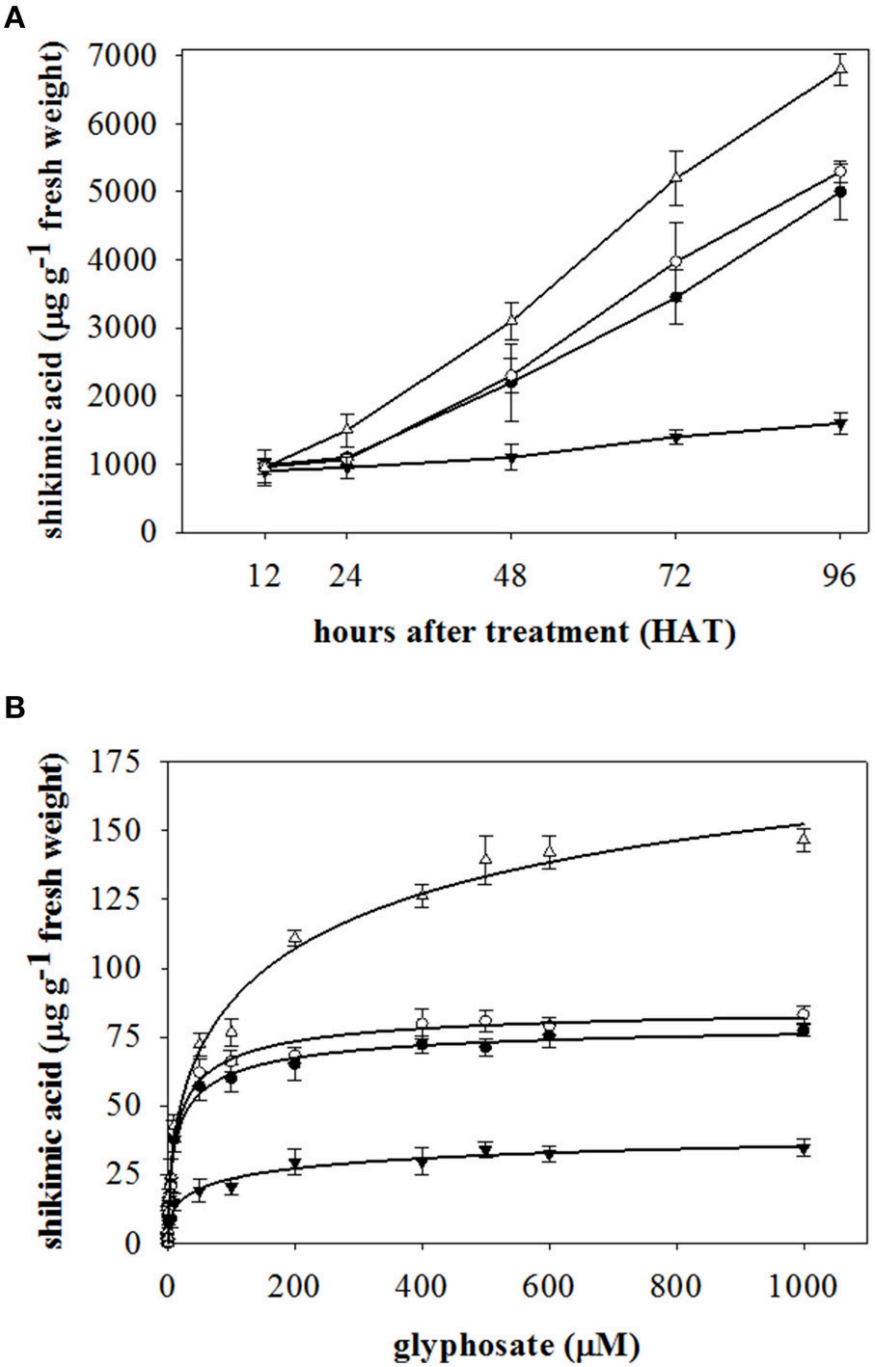
**Shikimic acid accumulation in (A) leaves of plant, and (B) leaf segments of plants from E (●) and UE (○) accessions of *A. sterilis*, and R (▼) and S (Δ) populations of *L. rigidum***. Vertical bars are ± standard errors of the mean.

In this study we compared the effect of glyphosate on two different species; two *A. sterilis* (E and EU) accessions, and two *L. rigidum* populations (R and S to glyphosate) as shown in Figures 2A,B.

Shikimic acid accumulation in both accessions of *A. sterilis* was similar with no significant differences at the different h. At 48 HAT, shikimic acid accumulation increased two times in each accession. Figures 2A,B shows that the difference in accumulation of R and S-glyphosate populations of *L. rigidum* can be compared with the accessions of *A. sterilis*. As previously described, the accumulation rate was observed by means of the slope of the linear regression of each population. The accumulation rate of R- glyphosate population of *L. rigidum* [*y* = 753.08 + 8.66 x; *R*^2^ = 0.99] was six-fold lower than the S- glyphosate population [−106.10 + 71.74 x; *R*^2^ = 0.98] of the same species. The accumulation rates for E and UE accessions of *A. sterilis* were similar, showing values of [85.67 + 48.81 x; *R*^2^ = 0.99] and [−20.36 + 54.38 x; *R*^2^ = 0.99], respectively (Figure 2A). The increase of shikimic acid accumulation observed by UE compared with E is probably related with the greater reduction in GR_50_ and LD_50_ values of UE accession (Table 1). The UE accession accumulated 1.07-fold more than E accession of *A. sterilis* when glyphosate application was 1000 μM (Figure 2B). However, the comparison between *L. rigidum* populations showed an accumulation 4.22 fold higher for S compared to R at the same glyphosate rate.

### ^14^C glyphosate absorption, translocation, and visualization

Accessions of *A. sterilis* showed no significant differences in ^14^C glyphosate absorption and translocation assays at different application times (Table 2). Across each accession and harvest times (HAT), ^14^C-herbicide absorption ranged from 12 to 76% of applied radioactivity (Table 2). Progressively, ^14^C-glyphosate translocated out of the treated leaf and into the other parts had similar radioactivity amount in both accessions. At 144 HAT, radioactivity recovered in treated leaves remained similar (76.52 to 75.34) in E and UE plants.

**Table 2:** **^14^C glyphosate absorption and translocation in two accessions of *A. sterilis* at different HAT**.

**Accession**	**HAT**	**Absorption (% recovered)**	**Translocation (%^14^C absorbed)**		
			**Treated leaf**	**Rest of shoots**	**Roots**
E	12	15.29±2.61 g	86.32±2.69 a	13.50±1.65 f	0.18±0.06 g
	24	25.14±1.96 f	80.67±3.42 b	15.29±1.93 ef	4.04±1.23 fg
	48	49.93±4.15 d	74.22±5.18 c	17.39±2.08 d	9.39±2.11 de
	72	61.13±3.33 c	64.41±6.38 e	16.82±1.23 de	18.77±3.76 c
	96	70.38±4.68 b	60.69±3.93 ef	19.91±3.04 c	19.40±2.81 bc
	144	76.52±3.87 a	48.38±4.95 g	25.63±2.85 a	25.99±3.92 a
UE	12	12.66±1.09 g	90.09±4.96 a	9.11±1.82 g	0.80±0.17 g
	24	31.82±4.32 e	76.18±3.35 c	16.32±2.39 de	7.50±1.38 ef
	48	47.39±5.21 d	68.36±6.89 d	19.41±2.48 c	12.22±2.07 d
	72	59.42±3.94 c	59.72±5.88 f	21.79±3.14 b	21.49±3.27 bc
	96	74.07±5.17 ab	60.39±4.76 f	20.27±2.91 bc	19.34±2.14 bc
	144	75.34±4.86 a	51.87±3.74 g	23.90±3.01 a	24.23±3.66 ab

Mean value± standard error of the mean. Means on a same column followed by the same letter were not significantly different atα = 0.05. E, exposed to glyphosate applications; UE, never exposed to glyphosate applications.

**Figure 3:**
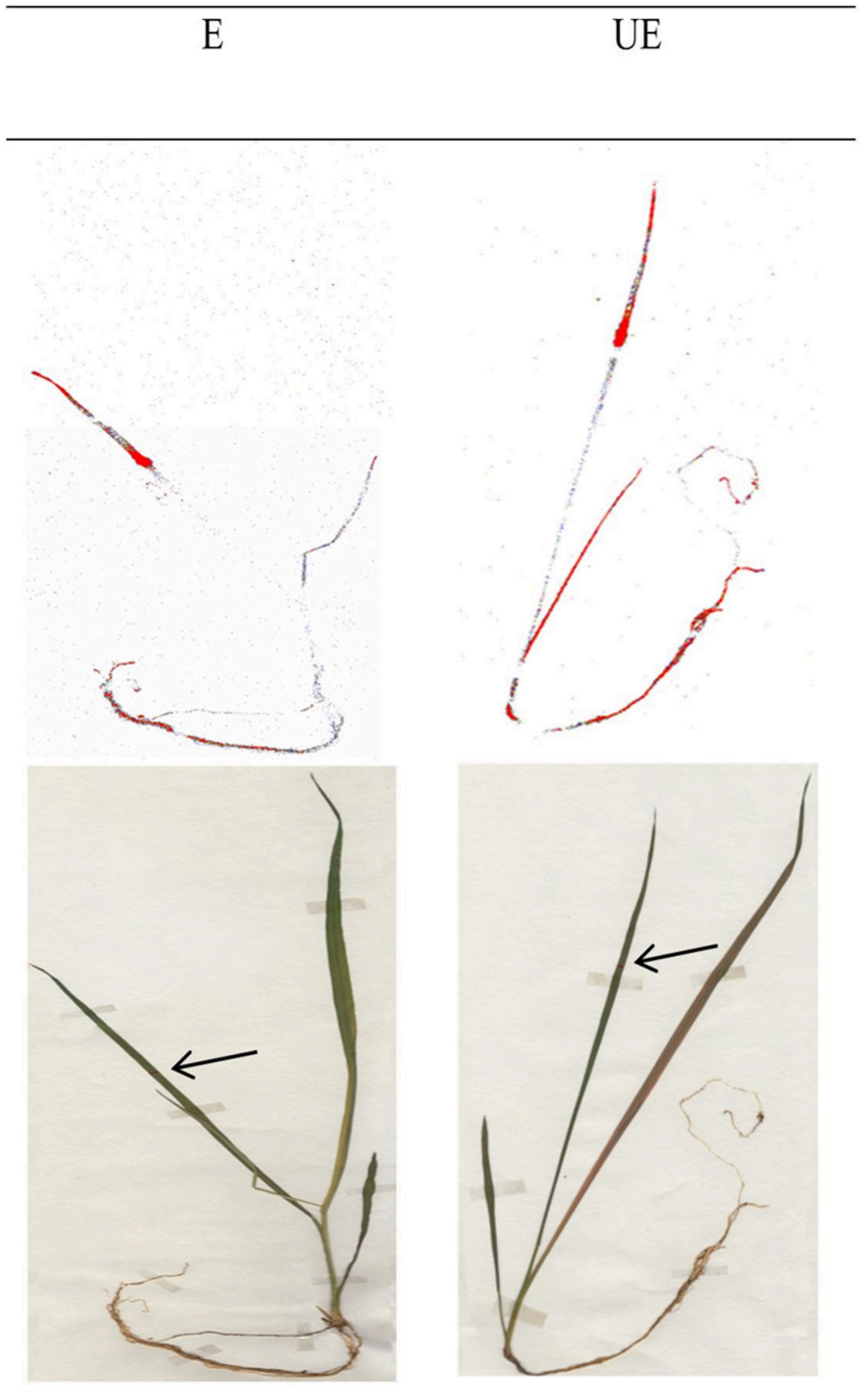
**Phosphorimaging visualization of ^14^C glyphosate translocation in E and UE accessions of *A. sterilis* at 144 HAT**. Intensity in the coloration shows greater herbicide rate. Plants have a BBCH 14-15 stage.

The ^14^C glyphosate visualization was similarly distributed between leaves, the rest of the shoot and the root with an acropetal and/or basipetal glyphosate movement in both E and UE accessions at 144 HAT (Figure 3).

### Metabolism study

**Figure 4:**
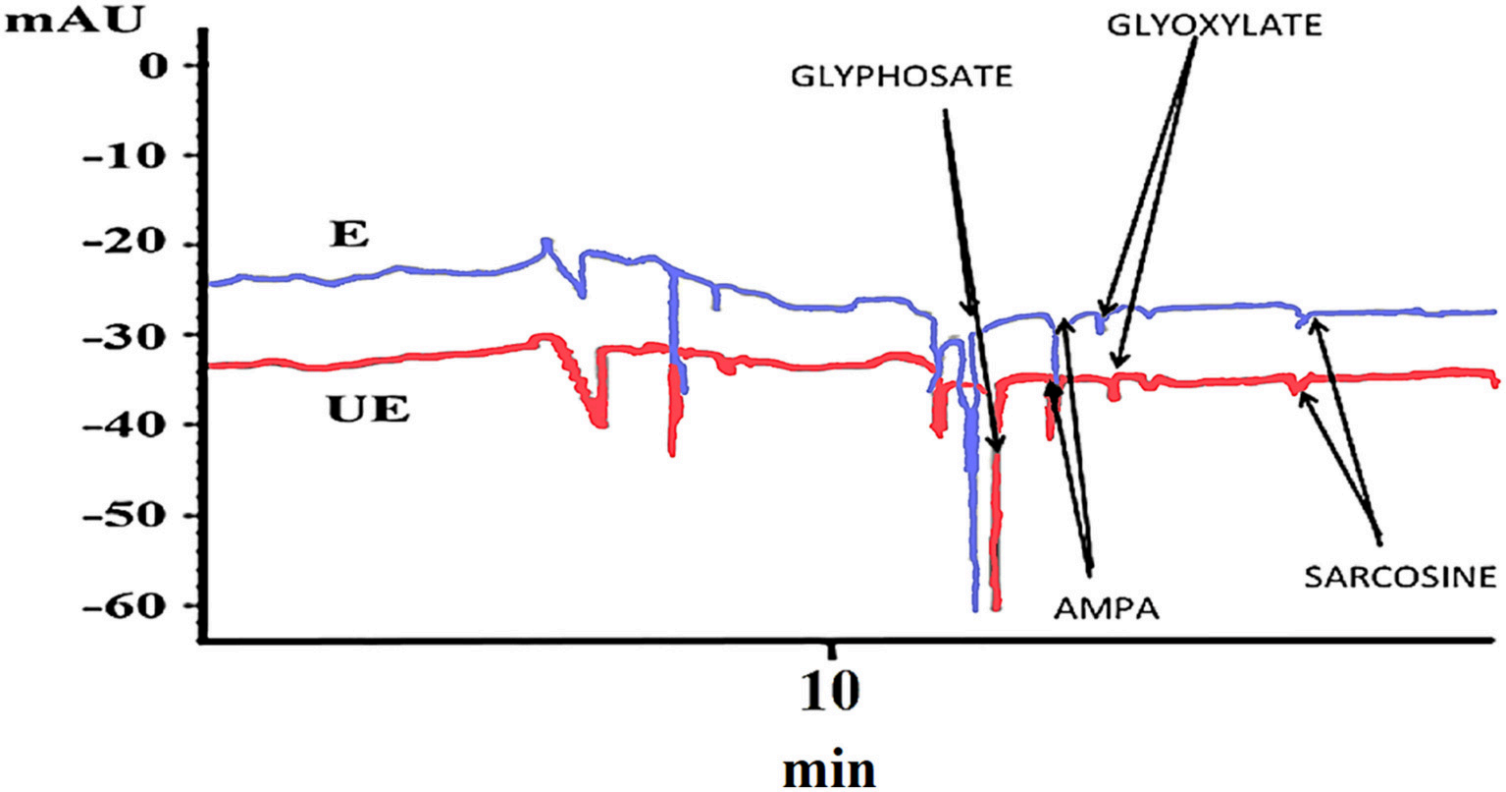
**Electropherogram of *A. sterilis* E plants treated with 300 g ae ha^−1^ of glyphosate at 144 HAT**.

This study shows that metabolism is slow but existent in both accessions of *A. sterilis*. At 96 HAT, AMPA starts to appear at low concentrations. At 144 HAT, the other metabolite (sarcosine) appears, but also in low concentrations (Figure 4). A factorial experiment or test with two parameters, accessions and metabolites, at 144 HAT, was carried out and analyzed by mean of ANOVA. The interaction between the two studied parameters was not significant. Less than 55% of glyphosate in relation to its metabolites (AMPA, glyoxylate and sarcosine) was detected in both accessions of *A. sterilis* (Table 3). These results show that the mechanism of the metabolism is involved in the innate tolerance of *A. sterilis* to glyphosate.

**Table 3:** **Glyphosate metabolism expressed as a percentage of total glyphosate and their metabolites in E and UE accessions of *A. sterilis* at different HAT glyphosate at 300 g ae ha^−1^ in the BBCH 13-14 stage**.

**Times HAT**	**Accession**	**Glyphosate**	**AMPA**	**Glyoxylate**	**Sarcosine**
12	UE	99.75± 3.12 a	ND	0.26± 0.02 b	ND
	E	99.68± 2.03 a	ND	0.31± 0.02 b	ND
24	UE	99.86± 2.10 a	ND	0.12± 0.01 b	ND
	E	99.79± 2.85 a	ND	0.20± 0.02 b	ND
48	UE	89.57± 3.64 b	8.99± 2.05 c	0.42± 0.03 b	ND
	E	88.66± 2.79 b	9.21± 1.92 c	0.30± 0.01 b	ND
96	UE	68.64± 1.93 c	30.91± 3.40 b	1.55± 0.62 a	ND
	E	67.48± 4.45 c	30.22± 2.03 b	1.39± 0.51 a	ND
144	UE	54.43± 2.96 d	40.63± 3.00 a	1.68± 0.47 a	5.01± 0.22 a
	E	55.98± 3.25 d	39.71± 1.89 a	1.74± 0.51 a	4.86± 0.37 a

Mean values± standard error of the mean. Means on a same column followed by the same letter were not significantly different atα = 0.05. E, exposed to glyphosate applications; UE, never exposed to glyphosate applications.

### EPSPS enzyme activity

**Figure 5:**
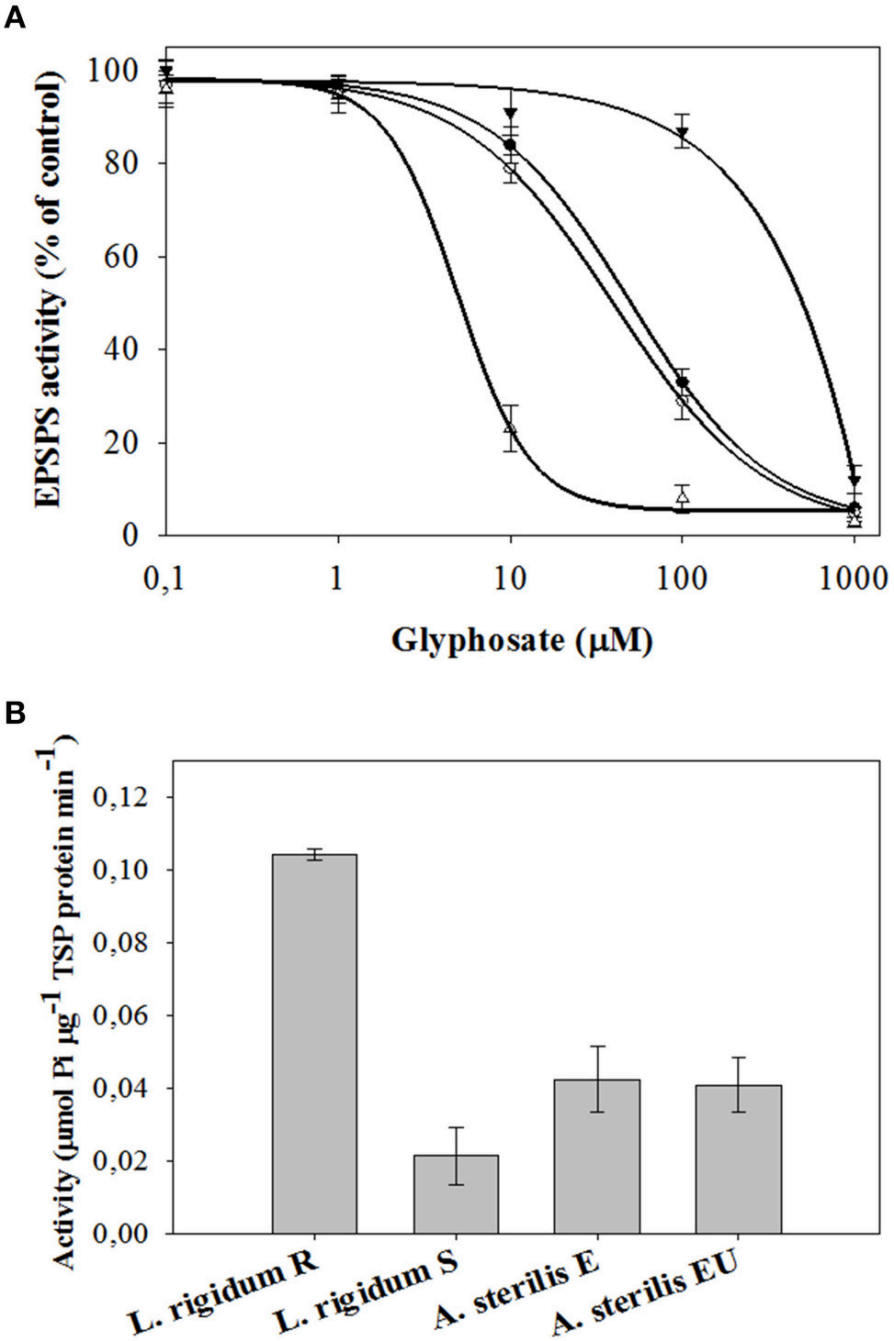
**(A)** EPSPS enzyme activity expressed as percentage of the untreated control in leaf extracts of plants from E (●) and UE (○) accessions of *A. sterilis*, and R (▼) and S (Δ) populations of *L. rigidum*. Vertical bars are ± standard errors of the mean. **(B)** Basal EPSPS activity for E and UE accessions of *A. sterilis*, and R and S populations of *L. rigidum*. Vertical bars are ± standard errors of the mean (*n* = 3).

As shown in Figure 5A, the EPSPS enzyme activity from all species, populations and accessions was inhibited by glyphosate. However, the EPSPS values between them were different (Table 4). The EPSPS activity from the *L. rigidum* R presented an I_50_ (400.02 μM) 78.5 times higher than that of the S population. This value was also higher (but not so high) than the presented by E and UE accessions of *A. sterilis.* In this last case the I_50_ values for both accessions of *A. sterilis* were 53.51 and 42.41 μM for E and UE (*P* = 0.395), respectively (Table 4). These results did not reveal any differences between both accessions respect to the EPSPS activity. The same trend was found in “dose- response assays” and in “shikimic acid accumulation” studies.

**Table 4:** **Parameter estimates of the equation used to calculate the sensitivity of EPSPS enzyme activity to glyphosate in extracts from leaf tissue of plants from *L. rigidum* populations and *A. sterilis* accessions**.

**Species**		**c**	**d**	**b**	**I_50_(μM)(95% CI)^a^**	**RI**	***P***
*L. rigidum*	R	1.23	97.17	1.57	400.02 (457.51–342.53)	78.43	<0.0001
	S	1.54	98.18	1.99	5.10 (6.19–4.01)		
*A. sterilis*	E	0.60	98.37	1.03	53.51 (61.40–45.62)	1.26	0.395
	UE^*^	1.12	98.70	0.95	42.41 (50.82–34.00)		

* Considered in this experiment as susceptible.

a CI values are the 95% confidence intervals (n=3).

E, exposed to glyphosate applications; UE, never exposed to glyphosate applications.

The specific activity of EPSPS in the absence of glyphosate (basal enzyme activity) was 0.104, 0.023, 0.0431, and 0.0412 μmol μg^−1^ protein min^−1^ for *L. rigidum* R, *L. rigidum* S, *A. sterilis* E, and *A. sterilis* UE, respectively (Figure 5B). The R plants of *L. rigidum* showed five times higher basal EPSPS enzyme activity than the S plants. In the case of both accessions of *A. sterilis* no significant differences were observed between them.

## Discussion

Cover crops have helped to reduce the soil erosion in Mediterranean perennial crop areas, mainly in olive groves (*Olea europea* L.). However, during the dry period the weeds have to be controlled before they start to compete with the main crop for water and nutrients. As previously mentioned one of the best weeds used as cover crops is *A. sterilis* which has a life cycle from winter to summer and has to be controlled in spring mainly with glyphosate herbicide (De Prado et al., 2012). During the last five year farmers have noted that glyphosate has never been a good control of *A. sterilis*. However, the glyphosate control of other monocot and dicot weeds was adequate. Trying to answer this question we carried out a study on two *A. sterilis* accessions, one never exposed with glyphosate (UE), and another exposed during a five year period (E). The results obtained in a growth chamber showed a high GR_50_ (dose causing 50% reduction in fresh weight) in both accessions (245.2 to 297.1) and it is also very important to note that LD_50_ (the dose causing 50% mortality) in both accessions was lower than the field dose used by farmers. The results show an innate tolerance to glyphosate and, despite successive applications carried out over 5 years; their resistance has not evolved. Supporting these results other researchers studying different glyphosate-tolerant species have shown GR_50_ values of 351.01, 403.82, 362.94, 600.28, and 175.30 g ae ha^−1^ for *Canavalia ensiformis, Mucuna pruriens, Neonotonia weightii, Clitoria ternatea*, and *Cologania broussonetii*, respectively (Cruz-Hipólito et al., 2009, 2011; Rojano-Delgado et al., 2012; Alcantara-de la Cruz et al., 2016).

Several studies have shown that spray retention assays are important in order to evaluate and compare herbicide efficacy among weed species. In previous spray retention studies, higher values were obtained for both populations of glyphosate-resistant grass weeds, such as *Lolium multiflorum* (Michitte et al., 2007) and *Leptochloa virgata* (Pérez-López et al., 2014). However, it has been reported that it is rather difficult to find any resistance/tolerance between populations within a species due to a reduction in spray retention of the herbicide, as occurred in the present study (Michitte et al., 2007; Nandula et al., 2008; Cruz-Hipólito et al., 2011; Pérez-López et al., 2014; De Prado et al., 2016). The results could be attributed to the similarity of the cuticle of the leaves of both accessions (Shepherd and Griffiths, 2006; Heredia-Guerrero et al., 2014).

It is widely known that shikimic acid accumulation in weed extracts is the result of EPSPS inhibition due to foliar application of glyphosate (Amrhein et al., 1980; Shaner et al., 2005). Such inhibition has been accepted as an indicator of the sensitivity of the weeds to the herbicide. The relative values instead of findings revealed in shikimic acid accumulation from both E and UE accessions of *A. sterilis* were intermediate to those obtained in *L. rigidum* populations. This shows that the low accumulation of shikimic acid is characteristic of R-glyphosate populations. On the contrary, a high shikimic acid accumulation is observed in S-glyphosate population and an intermediate accumulation in glyphosate-tolerant species (Singh and Shaner, 1998; Cruz-Hipólito et al., 2011; Rojano-Delgado et al., 2012; Alarcón-Reverte et al., 2013). The intermediate shikimic acid accumulation found in E and UE accessions of *A. sterilis* was similar to the level of tolerance to glyphosate found in dose-response assays.

These results suggest that the two accessions of *A. sterilis* have an innate tolerance to glyphosate regardless of the former applications that farmers have made to control this weed.

Addition, these results are in agreement with those obtained in tolerant legume weeds, which showed a lower ^14^C glyphosate absorption and translocation than other broadleaf weeds susceptible to glyphosate (Cruz-Hipólito et al., 2009, 2011; Rojano-Delgado et al., 2012; Alcantara-de la Cruz et al., 2016). Foliar absorption and subsequent glyphosate translocation in the plant are two parameters directly related to the biological effectiveness of the herbicide. The reduction in one parameter or both of them has contributed to explaining the glyphosate resistance between populations of the same species. In the present study, the ^14^C-glyphosate absorption and translocation values found in E and UE accessions of *A. sterilis* were similar. As in other tolerant species studied to date, it was observed that glyphosate moves in both directions within the plant (via xylem and phloem) i.e., glyphosate has an amphimobile behavior in *A. sterilis* as previously shown by McAllister and Haderlie (1985) and Menendez et al. (2014). These results suggest that the two accessions of *A. sterilis* have an innate tolerance to glyphosate regardless of the former applications that farmers have made to control this weed.

For the first time, the oxidase activity of glyphosate (GOX) was detected in extracts from soybean cell cultures capable of degrading the herbicide to less toxic metabolites (AMPA, glyoxylate, and sarcosine). However, degradation was rather inadequate to explain resistance or tolerance in plants (Feng et al., 2004; Cruz-Hipólito et al., 2009, 2011; Gonzalez-Torralva et al., 2012; Alarcón-Reverte et al., 2015; Ribeiro et al., 2015). Nevertheless, in the last 5 years, other authors have shown the existence of species with resistant populations capable of degrading glyphosate to non-toxic metabolites (AMPA, glyoxylate, sarcosine and formaldehyde), whereas susceptible populations lack this possibility (De Carvalho et al., 2012; Gonzalez-Torralva et al., 2012). In addition, a legume *M. pruriens* var. *utilis* has been found to be tolerant with a high capacity to metabolize glyphosate by means of glyphosate oxidase (Rojano-Delgado et al., 2012). The results show that the mechanism of the metabolism is involved in the innate tolerance of *A. sterilis* to glyphosate.

Normally, an increased glyphosate resistance (higher EPSPS enzyme activity and lower shikimate accumulation) is associated with a greater EPSPS gene amplification, EPSPS transcript levels, EPSPS protein expression, and/or genomic copy number (Baerson et al., 2002; Salas et al., 2012, 2015; Mao et al., 2016). According to Salas et al. (2012), mutations in the catalytic site of the EPSPS in plants from natural populations are unusual, and in fact, it is the most conserved part. The glyphosate selection pressure favors the survival of individuals with multiple copies of the glyphosate target gene. The higher amount of EPSPS per unit of a protein (and also per fresh weight) decreases the herbicide effect, allowing the shikimate pathway not to be blocked. Taking this into account, the differences between the populations of *L. rigidum* (R and S) could be explained by an increase in the EPSPS genomic copy number in the R compared to the S population (Alarcón-Reverte et al., 2015; Fernández et al., 2015). In the case of both accessions of *A. sterilis* (E and UE), the similarity in the EPSPS activity enzyme with different glyphosate concentrations and without glyphosate, shows that both populations present the same genomic characteristics. This is valid regardless of whether it is a matter of the EPSPS copy number, the overexpression or others, because there is no difference in the resistance mechanisms between both accessions of *A. sterilis.*

## Conclusions

The prospecting conducted showed homogeneous results between all accessions collected, and therefore, all these have the same level of innate tolerance to glyphosate. The above results indicate that non-target site mechanisms are involved in the innate tolerance to glyphosate in *A. sterilis.* This is probably partly due to a reduced herbicide absorption/translocation and glyphosate-metabolism. The results of the present study confirm that mechanisms based on changes of the site of action did not confer innate tolerance to glyphosate in *A. sterilis.* Moreover, the findings of the present study highlight the need of an integrated weed management approach without any exclusive reliance on specific herbicides such as glyphosate in order to keep the long-term efficacy of each involved method and ensure the sustainability of the system.

## Author contributions

PF, RA, and RD performed the glyphosate plant response; AR performed EPSPS activity assays; PF, RA, HC, IT, and RD performed ^14^C-glyphosate absorption/translocation, and visualization; AR performed ^14^C-glyphosate metabolism.

### Conflict of interest statement

The authors declare that the research was conducted in the absence of any commercial or financial relationships that could be construed as a potential conflict of interest.

## Abbreviations

AMPA: aminomethylphosphonic
EPSPS: 5-enolpyruvylshikimate-3-phosphate synthase
NTSR: non-target site resistance
NTST: non-target site tolerance
TSR: target site resistance
E: seeds exposed to glyphosate applications
UE: seeds never exposed to glyphosate applications
DAT: days after treatment
HAT: h after treatment
TI: Tolerance Index
RI: Resistance index
GOX: oxidase activity of glyphosate.
